# Analysis of Quadriceps Fatigue Effects on Lower Extremity Injury Risks During Landing Phases in Badminton Scissor Jump

**DOI:** 10.3390/s25082536

**Published:** 2025-04-17

**Authors:** Jun Wen, Datao Xu, Huiyu Zhou, Zanni Zhang, Liangliang Xiang, Goran Munivrana, Yaodong Gu

**Affiliations:** 1 Faculty of Sports Science, Ningbo University, Ningbo 315211, China; wennjunn666@gmail.com (J.W.); xudatao3@gmail.com (D.X.); zhouhuiyu@nbu.edu.cn (H.Z.); zhangzanni@gmail.com (Z.Z.); 2 KTH MoveAbility Lab, Department of Engineering Mechanics, KTH Royal Institute of Technology, 114 28 Stockholm, Sweden; 3 Department of Kinesiology of Sport, University of Split, HR-21000 Split, Croatia; goran.munivrana@gmail.com; 4 Faculty of Engineering, University of Szeged, 6720 Szeged, Hungary

**Keywords:** badminton, landing, fatigue, lower limb joint injury, lower limb stability, biomechanics

## Abstract

The scissor jump (SKJ) is vital in badminton, particularly for backcourt shots, but fatigue increases lower limb load and injury risk. This study investigates how quadriceps fatigue affects biomechanical characteristics and load during SKJ landing, aiming to understand its impact on injury risk. This study involved 27 amateur male badminton players from Ningbo University. Quadriceps fatigue was induced via knee exercises and footwork drills. Biomechanical data before (prior fatigue—PRF) and after fatigue (post fatigue—POF) were recorded using a force platform and motion capture system. Muscle activation was measured with EMG and analyzed through musculoskeletal modeling, with paired t-tests and SPM 1D (Statistical Parametric Mapping 1D) for statistical analysis. Under the POF condition, knee flexion angle increased, and power decreased (*p* < 0.001, *p* < 0.001, respectively); ankle plantarflexion angle increased, and power decreased (*p* < 0.001, *p* < 0.001, respectively). As fatigue progressed, joint reaction forces initially decreased but later increased. Joint energy dissipation decreased, with differences more pronounced in the coronal than sagittal plane. Achilles tendon force and anterior–posterior tibial shear force decreased, while coronal plane center-of-mass displacement increased. Findings show quadriceps fatigue harms limb stability, upping knee and ankle loads, disrupting the movement pattern, and risking coronal plane injuries. It is recommended that athletes enhance quadriceps endurance, improve neuromuscular control, and refine landing techniques to maintain stability and prevent injuries when fatigued.

## 1. Introduction

As a high-speed, high-skill, and highly competitive sport, badminton demands high physical fitness from its athletes [1]. During a match, the shuttlecock travels at very high speeds, requiring players to make rapid movements and precise shots in a short time. The fast-changing pace and diverse techniques require players to master various footwork patterns to reach positions quickly and strike the shuttle efficiently. Proper footwork not only improves shot efficiency but also conserves energy [2,3]. Among these footwork techniques, the scissor jump (SKJ) is frequently used in the backcourt. This technique involves a “scissor” motion with the legs in mid-air during a jump. Specifically, the non-racket side leg starts in front of the body before takeoff and rotates behind the torso in mid-air. The main advantage of the SKJ is that, while returning overhead shots, the player can quickly adjust their center of gravity through the crossing of their legs during the jump and generate additional power upon landing, helping them quickly return to the court’s center or front court. Statistics from the African Championships show that male singles players use the SKJ technique approximately 38.3 times per match [4], highlighting its importance and the need for further research.

The sudden stops and starts and frequent direction changes in badminton place high-intensity loads on the lower limbs. Previous studies have shown that the injury incidence in badminton is 1–4 injuries per 1000 h, with common injuries including strains, sprains, tendinopathies, and stress fractures [5]. Among these, anterior cruciate ligament (ACL) injuries, ankle sprains, and Achilles tendon injuries are the most common. The ACL is a crucial component of the knee joint that enhances stability, safeguards joint function, and contributes to overall movement efficiency [6]. However, ACL injuries primarily occur when the knee fails to stabilize the body’s center of gravity during SKJ landings [7], leading to excessive joint reaction forces from the abrupt deceleration. The ankle joint plays a central role in force transmission and shock absorption during the SKJ. It helps with explosive takeoff and protects the knee joint and overall body stability during landing. Ankle sprains may occur when the center of gravity shifts too much during a rapid landing, causing the ankle to exceed its normal range of motion [8]. The Achilles tendon, which connects the ankle joint to the calf, plays a critical role in supporting and transmitting forces. It is subjected to significant stretching and impact forces during jumping and landing [9]. In high-intensity badminton activities, the Achilles tendon may become injured due to over-fatigue or excessive force from sudden explosive movements [10]. Among these injuries, overuse injuries are most common, and lower limb injuries are prevalent in both professional athletes and amateur players. Additionally, previous studies have also indicated that fatigue increases the risk of these injuries occurring [11]. Therefore, studying the biomechanical characteristics of SKJ landing under fatigue conditions is crucial for developing strategies to prevent sports injuries.

One definition of exercise-induced fatigue is the decrease in force produced by the neuromuscular system during maximum voluntary contraction, and its impact varies depending on the sport [12]. Studies have shown that fatigue negatively affects proprioception, stability, and muscle state, leading to changes in an athlete’s posture, reaction time, and decision-making abilities [13,14,15]. In badminton, fatigue not only slows an athlete’s movement speed, reduces shot power, and decreases agility and balance but may also affect the athlete’s tactical decisions, shot accuracy, and judgment of shuttle placement. Fatigue in badminton affects the kinematic and dynamic parameters of the lower limbs, especially the ankle and knee joints [16]. Studies have confirmed that fatigue impacts joint angles and reaction forces at the ankle and knee during landing, compromising lower limb balance and stability [17,18]. Prior research has demonstrated that localized fatigue can affect the biomechanical characteristics of landing during single-leg vertical jumps [19,20]. However, to date, there has been little research on how quadriceps fatigue impacts the biomechanics of the SKJ landing. Specifically, during the initiation of the SKJ, the quadriceps contract concentrically to transfer force to the kinetic chain, assisting the athlete in completing the shot. Upon landing, the quadriceps then eccentrically contract to support the body’s descent and maintain stability. Included in the quadriceps muscle group are the vastus lateralis, vastus medialis, vastus intermedius, and rectus femoris, linking the thigh to the core while contributing to stability and strength [21]. On the other hand, when the quadriceps are fatigued, it puts additional stress on the knee joint, which may trigger compensatory adjustments in the ankle joint, ultimately impacting performance [22].

The center of mass (COM) refers to the point where the mass of a system is concentrated, and it can be used to analyze the trajectory of the body’s center of gravity during movement [23], helping to prevent injuries. During SKJ landing, fatigue also affects the distribution of the COM and the center of pressure in badminton participants. Impaired lower limb balance not only impacts the muscle force production and alters movement patterns but also potentially raises the likelihood of lower limb injuries [16]. Furthermore, some scholars have found that the change in COM height in male badminton players is greater than in female athletes, possibly due to differences in the force output between male and female athletes [24]. Thus, we consider COM as a key indicator to assess the impact of quadriceps fatigue on body stability.

We observe that previous studies have yet to investigate how quadriceps fatigue affects the biomechanical properties during the landing phase of badminton SKJ movements. Given that the quadriceps play a key role in the lower limb kinetic chain, understanding how quadriceps fatigue impacts the biomechanics of the SKJ landing, especially the ankle and knee joint changes, can help make strategic adjustments during sports. This research aims to investigate the effect of fatigue on lower limb injury risk during the landing of the badminton SKJ, focusing on how quadriceps fatigue influences biomechanical characteristics and load. The significance of this research is to provide recommendations for injury prevention strategies based on adjustments in lower limb joint movements after fatigue, and to refine training and rehabilitation strategies, aiming to enhance athletic ability while simultaneously lowering the probability of injury. We hypothesize that, in a fatigued state, the kinematic and dynamic parameters of the knee joint will directly change, while the ankle joint will undergo compensatory adjustments. Over time, as the body’s balance deteriorates, lower limb stability gradually declines, which in turn increases the load on the knee joint.

## 2. Materials and Methods

### 2.1. Participants

In this study, the required sample size was determined using G*Power software (version 3.1.9.7; Heinrich Heine University of Düsseldorf, Düsseldorf, Germany). A paired sample *t*-test with an effect size of 0.6, power of 0.8, and a statistical threshold of 0.05 was conducted for analysis of variance (ANOVA) [25]. The results indicated that in order to achieve a moderate effect size with a statistical threshold of 0.05, a minimum of 27 participants were needed. An aggregate of 27 male amateur badminton players (age: 22.8 ± 1.3 years; height: 175.88 ± 2.76 cm; weight: 67.86 ± 3.52 kg) were recruited from Ningbo University. Specific inclusion criteria were applied to ensure consistency: (1) All participants were healthy, young badminton enthusiasts from Ningbo University. (2) The participants played badminton at least three to four times a week, for at least two hours each time. (3) None had experienced a lower limb injury or any medical condition affecting the experiment within the past six months. (4) None had undergone lower limb surgery. An informed consent form was used to fully inform each participant of this study’s goals, methods, and requirements prior to data collection., which they carefully reviewed and signed. Ethical approval for the research was granted by the relevant committee (Protocol Code: TY 2025002).

### 2.2. Experimental Protocol and Procedures

**Figure 1:**
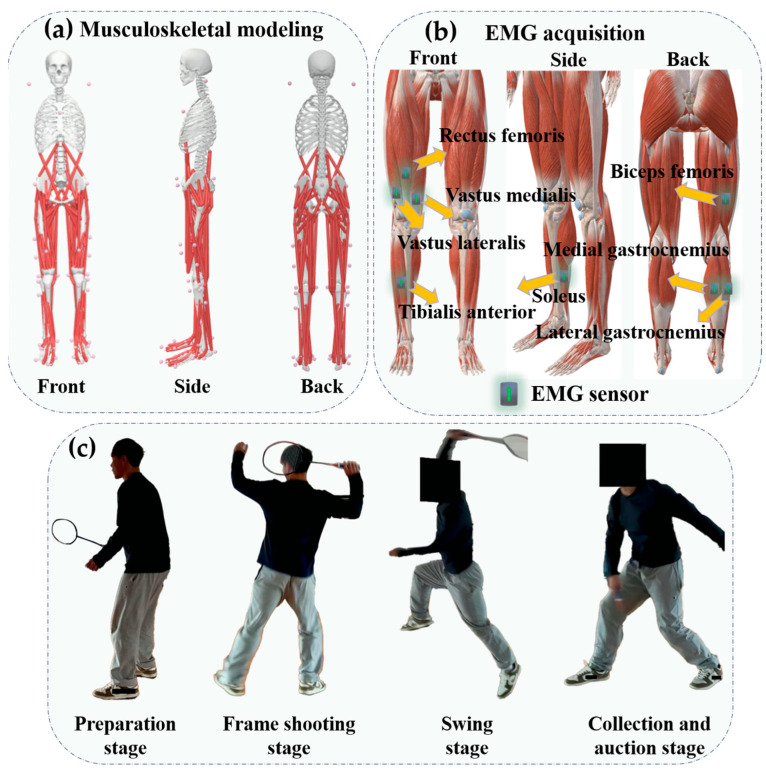
Schematic diagram of reflex markers, EMG positions, and action flow. (**a**) Illustration of the schematic representation of reflective marker placement on body skeletal landmarks. (**b**) Illustration of the position of an EMG test on a human lower limb. (**c**) Illustration of SKJ action process.

This research was carried out in the Sports Biomechanics Laboratory at Ningbo University. Prior to testing, essential participant details, including height and weight, were documented. Additionally, the length of the right leg (from the anterior superior iliac spine to the lateral malleolus) was measured to determine the initial positioning for movement execution during the experiment. This approach aimed to better simulate real-game conditions in badminton [4]. Following established protocols from prior research, 38 spherical reflective markers (12.5 mm in diameter) were strategically affixed to key anatomical sites on the participants’ bodies, ensuring precise motion tracking [26], as illustrated in Figure 1a. Motion data were captured using a Vicon system (Oxford Metric Ltd., Oxford, UK) equipped with eight high-speed cameras, each operating at a sampling frequency of 200 Hz. To gather kinetic data during the scissor jump (SKJ) landing trials, a force plate (AMTI, Watertown, MA, USA) was employed, sampling at 1000 Hz. The kinematic and kinetic data acquisition systems were fully synchronized to ensure accurate measurement. During the testing process, the subjects are required to wear the experimental clothing at all times. The Surface EMG for Non-Invasive Assessment of Muscles (SENIAM) recommendations were followed for applying electromyography (EMG) sensors. The soleus (SOL), medial gastrocnemius (MG), lateral gastrocnemius (LG), tibialis anterior (TA), rectus femoris (RF), vastus lateralis (VL), vastus medialis (VM), and biceps femoris (BF) muscle bellies were equipped with eight EMG sensors (Delsys, Boston, MA, USA) in order to measure muscle activation [27]. Sensor placements were carefully aligned with specific anatomical landmarks, as depicted in Figure 1b (adapted from Zhang, Xu et al. [28]).

Before the formal experiment, participants underwent a 15-min standardized warm-up session. The 15-min standardized badminton warm-up routine includes three parts: dynamic stretching, sport-specific activation, and cardiovascular activation. It covers full-body stretches for the neck, shoulders, hip joints, and other areas, as well as sport-specific movements like high knees, lateral slides, and simulated racket swings. Short sprints and multi-directional movements are incorporated to further elevate heart rate, fully activating muscles and joints in preparation for testing. This was followed by a series of simulated jump movements to maximize their performance potential during the experiment and reduce the risk of injury. Participants were then familiarized with the experimental environment and protocol, practicing the badminton scissor jump movement prior to the commencement of formal testing [4]. To reduce errors from differences in participants’ airborne times and landing distances, it was mandatory for all participants to ensure their test foot completely landed on the force platform during trials. If the test foot of the participant does not fully land within the force plate area, the test data will be unusable [18].

**Figure 2:**
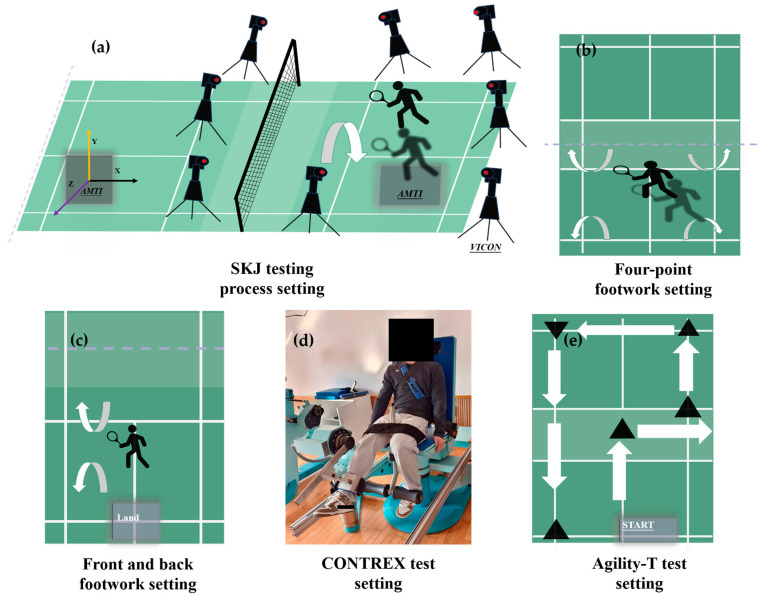
SKJ landing test and fatigue intervention flowchart. (**a**) Illustration of SKJ biomechanical test process. (**b**) Illustration of four-point footwork setting. (**c**) Illustration of front and back footwork setting. (**d**) Illustration of CONTREX isokinetic force gauge. (**e**) Illustration of agility-T test.

Pre-Fatigue Landings. Before the official experiment begins, a set of static data tests is conducted for static modeling in OpenSim. Following this, participants were instructed to perform a series of scissor jump movements, with five successful trials recorded. A successful trial was defined as landing on the non-racket side lower limb, as illustrated in Figure 2a. To prevent fatigue, an average rest interval of 45 s was provided between each scissors jump test. To standardize the impact load during the SKJ movements, participants began their jumps from a fixed distance in front of the force platform, corresponding to 50% of their leg length (the separation between the anterior superior iliac spine and the lateral malleolus). Additionally, to simulate badminton gameplay more realistically, participants were required to accelerate forward immediately after landing and reach a target located 3 m ahead of the force platform [4].

Post-Fatigue Landings. To realistically simulate the fatigue postures experienced during a badminton match, the fatigue protocol included 30 s of four-corner footwork, 30 s of front-and-back court footwork, quadriceps fatigue induction using an isokinetic dynamometer, and one set of the agility-T test [27]. First, before inducing fatigue, knee flexion and extension exercises were performed using a CONTREX isokinetic dynamometer (Contrex AG, Dubendorf, Switzerland), as shown in Figure 2d, to evaluate the knee extensor and flexor muscles’ maximal strength; three trials were conducted, and their average value was recorded. Second, participants were instructed to perform 30 s of the four-corner footwork drill on a badminton court while holding a racket, as shown in Figure 2b, followed immediately by 30 s of the front-and-back court footwork drill, as shown in Figure 2c. During the drills, participants were required to follow designated badminton footwork techniques in a specific sequence, with no rest between the drills. Third, immediately after the footwork drills, participants performed continuous knee flexion and extension exercises on the CONTREX isokinetic dynamometer at speeds of 60°/s and 120°/s for 3 min. Fatigue levels were evaluated based on the average torque produced during knee flexion [29]. A Rate of Perceived Exertion (RPE) scale from 6 to 20 was utilized to additionally evaluate participants’ subjective levels of fatigue [30]. Finally, to simulate real badminton match conditions, participants completed one set of the agility T-test immediately after the fatigue interventions, as shown in Figure 2e.

### 2.3. Data Processing and Analysis

The purpose of this study was to investigate how quadriceps fatigue affects lower limb biomechanical characteristics during the badminton SKJ landing phase. The initial point of contact was identified as the moment when the VGRF (vertical ground response force) exceeded 10 N. Data collection began at this moment and continued until the knee reached its peak flexion angle. Kinematic and kinetic data were extracted in C3D format using Vicon Nexus software (v1.8.5, Vicon Metrics Ltd., Oxford, UK) and later processed using MATLAB (R2022a, The MathWorks, Natick, MA, USA) through custom scripts to convert C3D files into TRC and MOT formats. Filter frequency selection was based on Winter’s prior research, and a selection of data was subjected to a dynamic residual analysis in order to improve the signal-to-noise ratio (SNR). A fourth-order zero-lag Butterworth low-pass filter was utilized, with cutoff frequencies set at 20 Hz for VGRF data and 10 Hz for kinematic data. The processed dataset was subsequently imported into OpenSim (v4.4, Stanford University, Stanford, CA, USA) for advanced biomechanical analysis and parameter computation. In OpenSim, a participant-specific static model was generated by scaling a generic musculoskeletal model according to individual body measurement [31]. Muscle origin and insertion sites were adjusted based on limb lengths to ensure anatomical accuracy. Joint kinematics were determined using OpenSim’s inverse kinematics tool, which produced motion files (MOT) capturing the scissor jump landing phase. To enhance kinematic precision, a dynamic residual reduction algorithm (RRA) was implemented to align the model with observed motion while optimizing the center-of-mass (COM) representation by minimizing residual forces in the frontal and transverse planes. Joint moments for each degree of freedom were calculated using inverse dynamics with a weighted least-squares optimization method, reducing discrepancies between experimental marker data and the computational model. Joint power was then determined by multiplying angular velocity by joint moments at each time frame. Muscle activation patterns were assessed through static optimization.

**Figure 3:**
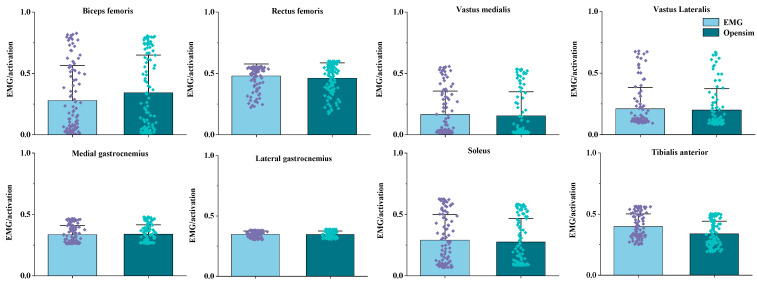
Illustration of EMG muscle activation, the light blue bar represents the EMG muscle activation results, while the dark blue bar represents the muscle activation results from the musculoskeletal model. The vertical scale ranges from 0 to 1, indicating muscle activation levels from none to full.

Signals from surface electromyography (EMG) were first band-pass filtered using a fourth-order Butterworth filter (10–400 Hz), then full-wave rectification and finally low-pass filtered with a cutoff of 6 Hz. The EMG signals were recorded at a sampling rate of 1000 Hz to ensure accurate capture of the signal within the frequency range of interest. EMG signal amplitudes were first normalized to the highest root mean square (RMS) value and then used maximal voluntary contraction (MVC) to further modify for comparative analysis of muscle activation levels. The accuracy and dependability of the model were assessed by comparing the musculoskeletal model simulations with the activation data from EMG sensors. Figure 3 shows that there were no statistically significant discrepancies between the simulated muscle activation levels and the observed EMG data.

Building upon previous studies, Achilles tendon force (ATF) is estimated by dividing the ankle plantarflexion moment by its corresponding moment arm. Earlier research designates the ankle plantarflexion angle as α. Prior investigations have employed magnetic resonance imaging (MRI) to determine the moment $M_{arm}$, which is derived using the following formula [32,33,34]:

(1) $$M_{arm}=-0.5910+0.08297\theta \alpha -0.0002606\theta^{2}\alpha$$

$M_{a}$ denotes the plantarflexion moment of the ankle joint, and Achilles tendon force (ATF) is computed using the equation below:

(2) $$ATF=\frac{M_{a}}{M_{arm}}$$

Joint reaction force ($F_{reaction}$) was calculated using the inverse dynamics tool in OpenSim [35]. It was determined based on the inputs of ground reaction force (GRF), joint kinematics, and segmental inertia into the Gait2392 model to compute the net reaction force at the ankle joint:

(3) $${\vec{F}}_{reaction}=-\left({\vec{F}}_{GRF}+\sum_{i}{\vec{F}}_{muscle}+m\vec{a}\right)$$

Tibial shear force (TSF) was calculated by decomposing the components of the knee joint reaction force. In this study, the knee joint reaction force ($F_{reaction}$) was obtained using the inverse dynamics tool in OpenSim. A system of local coordinates for knee joints was defined to determine the angle β between the direction of the reaction force and the plane of the tibial plateau [36]. The anterior–posterior tibial shear force was then calculated using the following formula:

(4) $$F_{shear}=F_{reaction}\cdot \sin\left(\beta \right)$$

### 2.4. Statistical Analysis

The Shapiro–Wilk test was performed to assess whether the data followed a normal distribution prior to statistical analysis. For normally distributed data, paired sample t-tests were utilized to compare key kinematic and kinetic parameters (e.g., joint angles, joint reaction forces, and energy dissipation) before and after quadriceps fatigue. To address the issue of multiple comparisons, a Bonferroni correction was applied to adjust the significance level for these paired *t*-tests, ensuring control of the family-wise error rate. To analyze time–series kinematic and kinetic data during the SKJ landing phase, Statistical Parametric Mapping (SPM) was employed. The obtained data were interpolated into 101-point time–series curves using a custom MATLAB algorithm, representing the entire landing phase from 0% to 100% [37]. Preprocessed data were analyzed using the open-source SPM1d script, which applies RFT-based corrections to address multiple comparisons in time–series data. All statistical computations were performed in SPSS 27.0 for Windows, with a significance level set at *p* < 0.05 [38]. Data visualization and graphing were completed using Origin 2022 software. The statistical analyses directly supported this study’s hypotheses. For example, one primary hypothesis was that quadriceps fatigue reduces lower limb stability. This was tested by comparing changes in knee and ankle joint angles, joint reaction forces, and center of mass (COM) before and after fatigue. Paired sample *t*-tests, with Bonferroni correction applied, revealed significant increases in knee flexion angles and decreases in ankle control following fatigue, indicating reduced lower limb stability. Additionally, SPM analysis, with RFT-based corrections, identified significant differences in time–series data during critical phases of landing, particularly in knee and ankle mechanics, further supporting this hypothesis.

## 3. Results

### 3.1. Joint Kinematics and Kinetics

**Figure 4:**
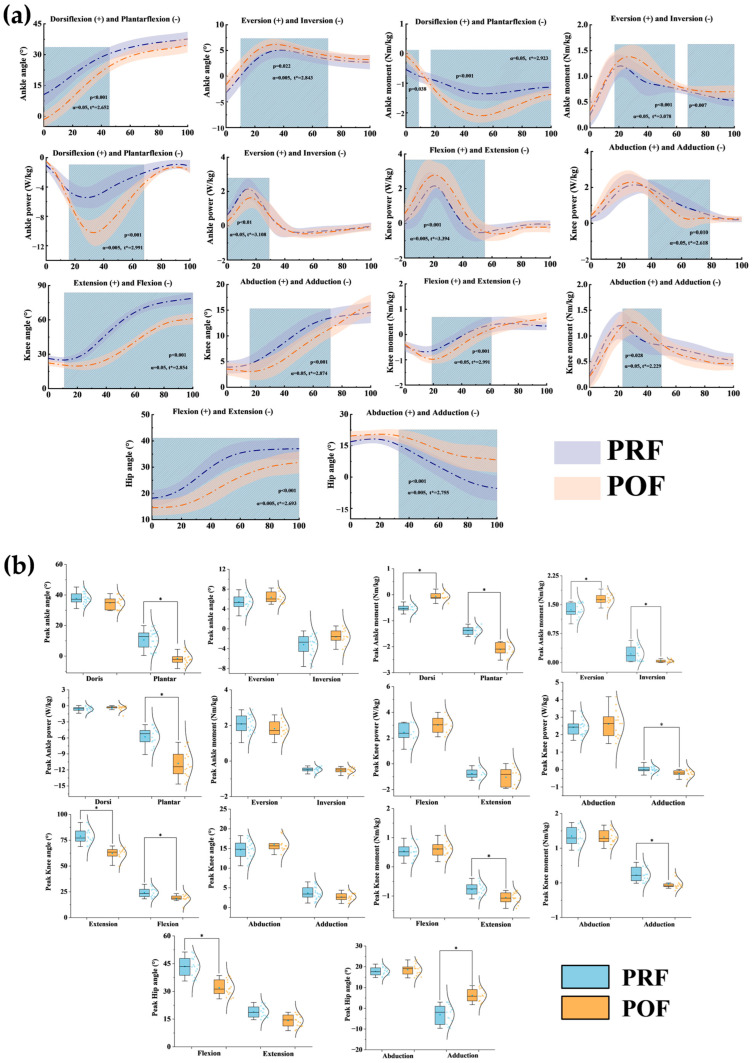
Influence of fatigue on kinematics, kinetics, and peak values during badminton scissor jump landing. (**a**) Shows SPM results for kinematic changes in the ankle, knee, and hip joints, as well as kinetic changes in the ankle and knee joints. Blue and orange lines represent pre- and post-fatigue conditions, respectively. (**b**) Highlights fatigue’s effects on kinematic and kinetic peaks in these joints. Blue and orange graphs indicate differences under pre- and post-fatigue conditions, with “*” marking significant regions above the threshold.

Figure 4a illustrates the kinematic differences during the landing phase of the badminton scissor jump before and after fatigue intervention. During the experiment, as fatigue developed, the participants’ ankle dorsiflexion angle decreased compared to the pre-fatigue condition, with significant differences observed in the 0–46% phase (*p* < 0.001). Additionally, the 10–70% phase saw an increase in the ankle eversion angle (*p* = 0.022). As fatigue progressed, the knee flexion angle rose significantly in the 11–100% phase (*p* < 0.001), and the knee abduction angle decreased significantly in the 18–71% phase (*p* < 0.001). In contrast, due to the effects of fatigue, the hip flexion angle decreased throughout the entire SKJ movement (*p* < 0.001). Furthermore, the hip abduction angle rose significantly in the 33–100% phase (*p* < 0.001).

Notable differences in joint angles between the PRF and POF conditions during the SKJ movement test are displayed in Table 1. Specifically, the plantarflexion angle of the ankle (*p* < 0.001), knee extension and flexion angles (*p* < 0.001 and *p* < 0.001, respectively), as well as the hip flexion, extension, and adduction angles (*p* < 0.001, *p* = 0.002, and *p* < 0.001, respectively), exhibited notable differences between the PRF and POF conditions.

Figure 4a illustrates the kinetic differences during the landing phase of the SKJ before and after the fatigue intervention. During the landing phase, the plantarflexion moment at the ankle joint increased in the 0–9% phase with the onset of fatigue (*p* = 0.038). Conversely, the plantarflexion moment decreased significantly in the 18–100% phase as fatigue deepened (*p* < 0.001). The ankle joint inversion moment increased during the 18–59% (*p* < 0.001) and 68–100% (*p* = 0.007) phases due to fatigue. For the knee joint, the extension moment decreased significantly in the 19–61% phase (*p* < 0.001), while the 22–50% phase saw a rise in the adduction moment (*p* = 0.028). During the landing phase of the SKJ, ankle joint plantarflexion power decreased in the 18–70% phase (*p* < 0.001), and ankle eversion power decreased significantly in the 0–30% phase (*p* < 0.001). In the knee joint, adduction power dropped with the progression of fatigue in the 39–79% phase (*p* = 0.010), while extension power rose significantly in the 0–55% phase (*p* < 0.001).

Table 2 shows significant differences in kinetic parameters throughout the SKJ’s landing phase. Specifically, significant differences were observed in the ankle dorsiflexion moment (*p* < 0.001); plantarflexion, eversion, and inversion moments and powers (all *p* < 0.001); as well as the knee flexion moment (*p* < 0.001), adduction moment (*p* < 0.001), and adduction power (*p* = 0.005)

### 3.2. Muscle Activation

**Figure 5:**
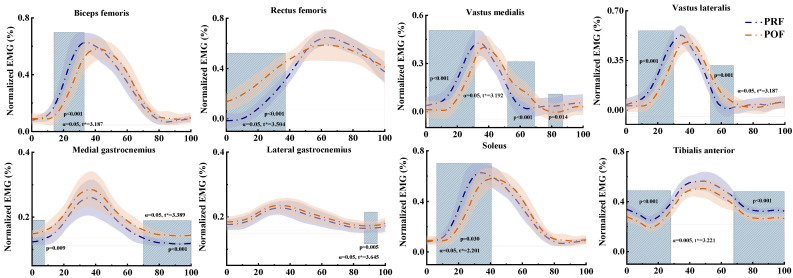
Mean ± SD normalized muscle activation during the SKJ. The figure shows SPM results before and after fatigue intervention. Blue and orange lines represent pre- and post-fatigue conditions, respectively, with shaded areas indicating regions of difference. The symbol “*” marks significant regions above the threshold.

Muscle activation during the SKJ landing before and after fatigue management is shown in Figure 5. When participants performed the SKJ experiment, BF activation significantly dropped in the 14–32% phase with the progression of fatigue (*p* < 0.001). RF activation rose significantly in the 0–38% phase with the progression of fatigue (*p* < 0.001). With the progression of fatigue, vastus medialis activation declined in the 2–31% phase (*p* < 0.001), elevated in the 52–79% phase (*p* < 0.001), and dropped again in the 78–97% phase (*p* = 0.014). Vastus lateralis activation reduced significantly in the 8–29% phase (*p* < 0.001) and elevated in the 53–68% phase (*p* = 0.001). Medial gastrocnemius activation rose significantly in the 0–8% (*p* = 0.009) and 70–100% (*p* < 0.001) phases with the progression of fatigue. Lateral gastrocnemius activation also rose, showing significant differences in the 88–95% phase (*p* = 0.005). Soleus activation lessened in the 7–41% phase with the progression of fatigue (*p* = 0.030). Tibialis anterior activation dropped, showing significant differences in the 0–28% and 68–100% phases (*p* < 0.001, *p* < 0.001, respectively).

### 3.3. Center of Mass, Energy Dissipation, Achilles Tendon Force, and Joint Reaction Force

**Figure 6:**
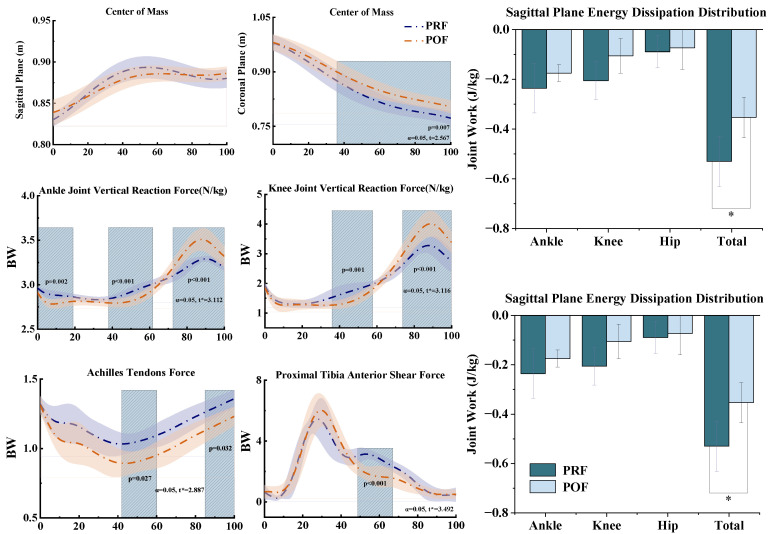
Effects of fatigue intervention on COM position, joint energy dissipation, Achilles tendon force, tibial shear force, and joint reaction force during SKJ landing. The figure shows SPM results pre- and post-fatigue. Blue and orange lines represent pre- and post-fatigue conditions, respectively, while dark and light blue bars indicate joint energy dissipation before and after fatigue. Shaded areas highlight differences, and “*” marks significant regions above the threshold.

Figure 6 illustrates the results for the center of mass (COM), energy dissipation, Achilles tendon force, and joint reaction force under fatigue conditions. For COM position distribution, no significant difference was observed in COM height changes as fatigue developed. However, the COM displacement distance increased significantly in the 37–100% phase (*p* = 0.007). For joint energy dissipation, in the sagittal plane, the total joint energy dissipation reduced markedly with the onset of fatigue (*p* < 0.001). In the frontal plane, energy dissipation at the ankle joint increased significantly compared to pre-fatigue conditions (*p* < 0.001). For Achilles tendon force, the SPM analysis indicated that Achilles tendon force decreased with the onset of fatigue, with significant differences observed in the 42–60% (*p* = 0.027) and 85–100% (*p* = 0.032) phases. For tibial shear force, SPM analysis revealed that tibial shear force decreased significantly in the 49–68% phase as fatigue deepened (*p* < 0.001). For ankle joint reaction force, SPM analysis showed that ankle joint reaction force reduced significantly in the 0–19% (*p* = 0.002) and 39–61% (*p* < 0.001) phases under fatigue intervention. However, as fatigue progressed, ankle joint reaction force increased significantly in the 72–100% phase (*p* < 0.001). Regarding knee joint reaction force, SPM analysis indicated a significant decrease in the 38–59% phase with the onset of fatigue (*p* = 0.001). However, knee joint reaction force rose significantly in the 73–100% phase (*p* < 0.001).

## 4. Discussion

This research aims to investigate the effect of fatigue on the risk of lower limb injuries during the landing of the badminton scissor jump (SKJ), with a focus on comparing the biomechanical differences in the lower limbs before (PRF) and after (POF) fatigue. We found that as fatigue develops, the center-of-mass (COM) displacement increases during the SKJ landing. This increase leads to instability in the lower limbs, characterized by side-to-side sway. Increased reaction forces on the ankle and knee joints impose greater stress on the lower limbs, and compensating changes in these joints make sports injuries more likely. This study’s primary contribution lies in its investigation of the biomechanics of the SKJ landing in badminton before and after fatigue. In contrast, previous studies have primarily focused on the biomechanics of movements such as lunges or smashes in badminton, with fewer studies on the SKJ action. Prior research on SKJ has primarily examined the differences in SKJ landings between amateur and professional male badminton players, analyzing kinematic, kinetics, and Achilles tendon forces [4]. Our study, however, provides a more comprehensive analysis by incorporating indicators such as kinematic analysis, kinetic analysis, muscle activations, and COM displacements.

Our initial hypothesis was that, in a fatigued state, the kinematic and kinetic parameters of the knee joint would change directly. In contrast, the ankle joint would undergo compensatory adjustments. Over time, as balance deteriorates, lower limb stability progressively diminishes, thereby imposing greater stress on the knee joint. Our findings validated this hypothesis, revealing significant differences in the angles of the ankle, knee, and hip joints under POF conditions compared to PRF. Previous studies have demonstrated that fatigue increases ankle dorsiflexion and knee flexion while decreasing hip flexion during jump landings [39]. In our study, however, we observed the opposite: under POF conditions, ankle plantarflexion and hip extension increased (*p* < 0.001, *p* < 0.001), while knee flexion increased (*p* < 0.001) (Figure 4a), likely aimed at mitigating impact forces and enhancing body stability [40]. Previous studies have shown that during a lunge landing in badminton, there is an increase in ankle eversion and knee abduction, with a reduction in hip abduction [41]. Our study found that as fatigue progressed, ankle eversion and hip abduction increased, while knee abduction decreased (*p* = 0.022 and *p* < 0.001, *p* < 0.001, respectively) (Figure 4a). This may be an attempt to maintain stability during landing under fatigue, with compensatory responses from the knee joint to reduce abduction [42,43].

Previous research has shown that fatigue during lunge movements increases knee flexion and abduction moments [29]. This finding aligns with our results, which indicate that fatigue during SKJ leads to an increase in knee flexion and abduction moments (*p* < 0.001 and *p* = 0.028). We detected a significant increase in plantarflexion and eversion moments in the ankle joint (*p* < 0.001 and *p* < 0.001) (Figure 4a). These findings suggest that fatigue may result in compensatory adjustments in the ankle joint’s moment output during lower limb landing. This adjustment may potentially worsen fatigue and impose greater stress on the joints. Previous studies have shown that, when comparing elite and amateur male badminton players performing similar movements, elite athletes exhibit reduced ankle joint power while demonstrating increased knee joint power, thereby distributing the impact force more evenly across the ankle, knee, and hip [4,44,45,46]. Similarly, our study demonstrated that as fatigue developed, plantarflexion power in the ankle joint decreased (*p* < 0.001), while knee flexion power increased (*p* < 0.001), and adduction power decreased (*p* = 0.010) (Figure 4a). This suggests that increased work in the sagittal plane during landing may lead to the knee joint bearing a greater load, thereby increasing the risk of knee injuries.

Previous research has demonstrated that jump landings increase the forces acting on the ankle and knee joints [21,47]. In our study, we focused on joint reaction forces. We detected a significant change in reaction forces during ankle and knee joint landing. Specifically, these forces initially decreased and subsequently increased (*p* < 0.001 for both) (Figure 6). This phenomenon can likely be explained by the reduced activation of the quadriceps and gastrocnemius muscles during the early stages of fatigue, which diminishes control over the ankle and knee joints, thereby resulting in lower reaction forces. As fatigue progressed, compensatory mechanisms engaged additional muscles to enhance stability [48], leading to an increase in reaction forces during the later stages of the movement. The sudden rise in loads on the knee and ankle joints could elevate the risk of sprains [49]. Notably, energy dissipation in the ankle joint’s coronal plane increased significantly (*p* < 0.001) (Figure 6). This suggests that fatigue-induced side-to-side sway became more pronounced, prompting compensatory adjustments in the ankle joint’s moment output during landing. These adjustments may exacerbate fatigue and further increase joint loading. This finding could indicate that fatigue reduces power output in the ankle joint, while the knee joint compensates by exerting greater force [50], potentially reducing the likelihood of ankle injuries [51]. Additionally, we found that as knee flexion increased, flexion power also increased significantly (*p* < 0.001), while energy dissipation at the knee joint decreased and joint reaction forces increased (*p* < 0.001). This suggests that increased work in the sagittal plane during landing may cause the knee joint to bear a greater load, compensating for the heightened effects of fatigue.

Notable differences were observed in muscle activation during SKJ landing between PRF and POF conditions. Previous studies have demonstrated that single-leg jumps lead to heightened activation of the vastus medialis, vastus lateralis, and rectus femoris following fatigue [18]. In contrast, the activation of the soleus, medial gastrocnemius, and lateral gastrocnemius diminishes during lunges, potentially increasing the risk of ankle sprains [52]. In our study, as fatigue progressed, the decreased knee abduction range modified the muscle load during landing, leading to a significant reduction in the overall activation of the biceps femoris, soleus, and tibialis anterior (*p* < 0.001, *p* = 0.030, and *p* < 0.001, respectively) (Figure 5). However, the activation of the rectus femoris, gastrocnemius medialis, and gastrocnemius lateralis significantly increased overall (*p* < 0.001, *p* < 0.001, and *p* = 0.005, respectively) (Figure 5). This enhancement improves the ankle joint’s control over lower limb stability during landing and prepares for the next stroke [53]. At the beginning of the movement, the vastus lateralis and vastus medialis exhibited reduced activation, but their activity levels increased in the later stages. This unstable activation pattern could serve as a compensatory mechanism to cushion the impact force on the joints during landing and maintain lower limb stability, yet it may also increase the risk of knee and ankle sprains [48,54].

Our study investigated lower limb stability after fatigue. Under POF conditions, the center of mass (COM) in the sagittal plane showed no significant difference compared to pre-fatigue. This indicates that fatigue had no significant impact on COM height, with minimal changes in the vertical fluctuations of the lower limbs. This finding is consistent with the observed reduction in Achilles tendon force and tibial shear force (*p* = 0.027, *p* < 0.001) (Figure 6). The reduction in Achilles tendon force may result from diminished activation of the soleus, thereby impairing force transmission through the tendon [55]. The decrease in tibial shear force might arise from the fatigued quadriceps’ inability to effectively support the knee joint, influencing the mechanical interaction between the knee and tibia [56]. Previous studies have shown that increased COM displacement during jump landings leads to side-to-side sway [57,58,59]. This finding aligns with our results, showing that COM displacement increased with fatigue (*p* = 0.007) (Figure 6). This suggests that fatigue impaired the balance control of the landing leg, increasing side-to-side sway in the lower limbs and potentially contributing to ankle and knee sprains in the coronal plane.

However, this study has certain limitations. Firstly, the tests were conducted in a laboratory setting, and owing to ceiling height limitations, participants were only able to hold a badminton racket and did not hit the shuttlecock during SKJ movements, which might differ from actual badminton competition scenarios. Secondly, the participants in this study were amateur male university students. Previous studies have shown that male and female athletes exhibit different responses to badminton movements [60]. Future research could mitigate these limitations by conducting experiments in actual badminton court environments, expanding the sample to include athletes of different genders, ages, and skill levels, and by incorporating advanced technologies like portable EMG systems and 3D motion capture, thereby enhancing the ecological validity and representativeness of the data. Additionally, assessing muscle fatigue using methods like zero-crossing rate (ZCR) and frequency-domain EMG parameters (e.g., median frequency and mean power frequency) could offer deeper insights into fatigue mechanisms across genders and ages [61]. Future research could investigate the effects of fatigue on athletes of various genders and ages by integrating advanced EMG-based metrics, such as ZCR, to provide a more comprehensive assessment of muscle fatigue. Since we only examined the non-racket leg of the participants, future studies could investigate differences between the racket and non-racket legs, which could contribute to injury prevention research in badminton. Lastly, future research could focus on analyzing the effects of fatigue on foot pressure, ACL stress, and patellofemoral joint loading during SKJ landing, providing deeper theoretical insights for injury prevention in badminton and offering scientific support for enhancing athlete performance and developing personalized training and rehabilitation programs.

## 5. Conclusions

This study investigates how quadriceps fatigue impacts the risk of lower limb injuries during the landing phase of the badminton scissor jump (SKJ). It uncovers the biomechanical mechanisms underlying fatigue in badminton and offers scientific insights for athlete training and injury prevention. The findings reveal that quadriceps fatigue significantly compromises lower limb stability, as evidenced by increased knee flexion, reduced ankle control, altered joint reaction forces, and impaired energy dissipation. These changes elevate loads on the ankle and knee joints, disrupt movement patterns, and necessitate compensatory muscle recruitment, thereby heightening the risk of coronal plane injuries. To mitigate these risks, it is recommended that athletes incorporate targeted quadriceps endurance and neuromuscular control exercises into their training routines. Additionally, optimizing landing techniques to minimize excessive knee flexion under fatigue can enhance joint stability and reduce injury likelihood. Future research should explore gender- and age-specific differences in fatigue responses during SKJ movements, as well as their effects on foot pressure distribution, anterior cruciate ligament (ACL) strain, and patellofemoral joint loading. Such studies could provide valuable data to inform personalized training and rehabilitation strategies.

## Figures and Tables

**Table 1:** Detailed results of peak joint angles of subjects performing SKJ at PRF and POF.

Parameters	Peak Value	Before (Mean ± SD)	Fatigue (Mean ± SD)	*p*-Value	Effect Size
Ankle Angle (°)	Dorsiflexion	37.70 ± 3.69	34.73 ± 3.80	0.055	0.37
	Plantarflexion	10.80 ± 6.40	−1.59 ± 0.87	<0.001 *	0.80
	Eversion	5.66 ± 1.52	6.34 ± 1.07	0.146	0.25
	Inversion	−3.22 ± 2.06	−1.65 ± 1.72	0.072	0.38
Knee Angle (°)	Extension	78.94 ± 2.96	61.08 ± 2.71	<0.001 *	0.95
	Flexion	24.95 ± 5.41	19.74 ± 3.80	<0.001 *	0.49
	Abduction	14.88 ± 2.56	15.99 ± 1.77	0.147	0.24
	Adduction	3.37 ± 1.44	2.63 ± 0.97	0.143	0.29
Hip Angle (°)	Flexion	43.48 ± 5.08	31.91 ± 4.61	<0.001 *	0.77
	Extension	18.85 ± 3.10	14.70 ± 3.08	0.002 *	0.56
	Abduction	17.75 ± 1.90	18.24 ± 2.37	0.521	0.11
	Adduction	−3.12 ± 4.44	5.90 ± 2.77	<0.001 *	0.77

Note: “*” indicates a significant difference (*p* < 0.05) between PRF and POF for the SKJ phase.

**Table 2:** Detailed results of peak joint moment and power of subjects performing SKJ at PRF and POF.

Parameters	Peak Value	Before (Mean ± SD)	Fatigue (Mean ± SD)	*p*-Value	Effect Size
Ankle Moment (Nm/kg)	Dorsiflexion	−0.54 ± 0.16	−0.07 ± 0.15	<0.001 *	0.83
	Plantarflexion	−1.38 ± 0.16	−2.10 ± 0.24	<0.001 *	0.87
	Eversion	1.37 ± 0.18	1.63 ± 0.16	<0.001 *	0.61
	Inversion	0.22 ± 0.20	0.04 ± 0.03	0.001 *	0.53
Ankle Power (W/kg)	Dorsiflexion	−0.63 ± 0.52	−0.39 ± 0.45	0.169	0.24
	Plantarflexion	−5.80 ± 1.71	−10.79 ± 2.46	<0.001 *	0.76
	Eversion	2.48 ± 1.05	−0.49 ± 0.14	<0.001 *	0.89
	Inversion	1.82 ± 0.45	−0.53 ± 0.15	<0.001 *	0.96
Knee Moment (Nm/kg)	Flexion	0.50 ± 0.26	0.62 ± 0.26	0.224	0.22
	Extension	−0.75 ± 0.20	−1.06 ± 0.18	<0.001 *	0.63
	Abduction	1.35 ± 0.27	1.33 ± 0.21	0.77	0.63
	Adduction	0.22 ± 0.20	−0.08 ± 0.09	<0.001 *	0.70
Knee Power (W/kg)	Flexion	2.44 ± 0.73	2.95 ± 0.61	0.203	0.35
	Extension	−0.76 ± 0.39	−1.17 ± 0.70	0.191	0.34
	Abduction	2.50 ± 0.51	2.61 ± 0.78	0.66	0.08
	Adduction	0.03 ± 0.18	−0.25 ± 0.26	0.005 *	0.53

Note: “*” indicates a significant difference (*p* < 0.05) between PRF and POF for the SKJ phase.

## Data Availability

The data supporting this study’s findings can be obtained from the corresponding authors upon reasonable request.
